# The role of the immune system in early-onset schizophrenia: identifying immune characteristic genes and cells from peripheral blood

**DOI:** 10.1186/s12865-024-00618-y

**Published:** 2024-05-03

**Authors:** Zi Chen, Yuxue Li, Yao Gao, Xiaoxuan Fan, Xinzhe Du, Xinrong Li, Zhifen Liu, Sha Liu, Xiaohua Cao

**Affiliations:** 1 Department of Mental Health, First Hospital/First Clinical Medical College, Shanxi Medical University, Taiyuan, 030001 China; 2 Shanxi Provincial Key Laboratory of Artificial Intelligence Assisted Treatment for Mental Disorders, The First Hospital of Shanxi Medical University, Taiyuan, 030001 China

**Keywords:** Early-onset schizophrenia, Immune characteristic genes, Immune characteristic cells, Immune system

## Abstract

**Background:**

Early-onset schizophrenia (EOS) is a type of schizophrenia (SCZ) with an age of onset of < 18 years. An abnormal inflammatory immune system may be involved in the occurrence and development of SCZ. We aimed to identify the immune characteristic genes and cells involved in EOS and to further explore the pathogenesis of EOS from the perspective of immunology.

**Methods:**

We obtained microarray data from a whole-genome mRNA expression in peripheral blood mononuclear cells (PBMCs); 19 patients with EOS (age range: 14.79 ± 1.90) and 18 healthy controls (HC) (age range: 15.67 ± 2.40) were involved. We screened for differentially expressed genes (DEGs) using the Limma software package and modular genes using weighted gene co-expression network analysis (WGCNA). In addition, to identify immune characteristic genes and cells, we performed enrichment analysis, immune infiltration analysis, and receiver operating characteristic (ROC) curve analysis; we also used a random forest (RF), a support vector machine (SVM), and the LASSO-Cox algorithm.

**Results:**

We selected the following immune characteristic genes: CCL8, PSMD1, AVPR1B and SEMG1. We employed a RF, a SVM, and the LASSO-Cox algorithm. We identified the following immune characteristic cells: activated mast cells, CD4^+^ memory resting T cells, resting mast cells, neutrophils and CD4^+^ memory activated T cells. In addition, the AUC values of the immune characteristic genes and cells were all > 0.7.

**Conclusion:**

Our results indicate that immune system function is altered in SCZ. In addition, CCL8, PSMD1, AVPR1B and SEMG1 may regulate peripheral immune cells in EOS. Further, immune characteristic genes and cells are expected to be diagnostic markers and therapeutic targets of SCZ.

**Supplementary Information:**

The online version contains supplementary material available at 10.1186/s12865-024-00618-y.

## Introduction

Schizophrenia (SCZ) is a devastating psychiatric disorder with an unclear etiology and a lifetime prevalence of 1%. It is characterized by significant abnormalities in cognitive function, thinking, emotion, behavior and other aspects of mental activity, and leads to impairments in occupational and social function [1]. The prevailing consensus in the literature is that SCZ is influenced by the confluence of genetic and environmental factors [2]. Early-onset schizophrenia (EOS) has an age of onset of < 18 years. Compared to adult SCZ patients, EOS patients have a higher genetic load and are less affected by environmental factors [3]. The study of EOS is helpful in revealing the genetic factors and pathogenesis of SCZ.

The pathogenesis of SCZ has not been fully elucidated. However, some studies have suggested that dysregulation of the central nervous system’s (CNS) immune processes may be a pathogenesis of SCZ [4, 5]. In 1973, Toney and Peterson proposed that inflammation may be involved in the development of SCZ. Maternal infection during pregnancy, chronic infection and autoimmune abnormalities may cause abnormal immune system function in patients with SCZ [6]. Studies have also shown changes in cellular and humoral immunity in patients with SCZ [7–9].

In recent years, increasing evidence has supported the immune-inflammatory hypothesis of SCZ. A large-scale genetic study on SCZ reported 108 significantly associated gene loci, with many newly discovered loci focusing on genes associated with the immune system [10]. Previous research suggests that there are disorders of immune cells in the CNS and peripheral blood in SCZ [11–13]. In addition, prior studies by our research group indicate that immune characteristic genes and cells are disrupted in three brain regions: the hippocampus, the prefrontal cortex and the striatum [14].

Although SCZ is considered to be a devastating neuropsychiatric disorder that primarily occurs in the CNS, postmortem brain biomarkers in SCZ are associated with molecular changes in the circulatory system in the living body [15, 16], this implies that the brain, CNS and periphery are interconnected. Thus, peripheral blood biomarkers are useful for identifying certain processes in the brain. It has been suggested that SCZ may influence the regulation of gene expression and metabolism in the peripheral blood via cytokines and neurotransmitters. At the same time, some immune-related changes in the peripheral blood may also be related to SCZ [16]. The diagnosis of SCZ has traditionally relied on a diagnostic system centered on clinical symptoms, which is easily affected by clinician bias and subjectivity, and lacks sensitive, specific neuropsychiatric biomarkers [17]. Although SCZ is considered a neuropsychiatric disorder that occurs predominantly in the brain, some studies have identified several biological markers in the peripheral blood of patients with SCZ [18, 19]. In sum, we suggest that the expression of immune-related genes and cells in the peripheral blood of patients with SCZ may be used as biomarkers to diagnose and predict the treatment effect of EOS.

We aimed to identify the immune characteristic genes and cells of EOS through a whole-genome mRNA expression array in the peripheral blood of EOS, and to determine the possible pathogenesis and biomarkers in the peripheral blood that may be used to diagnose and predict the treatment effects for cases of EOS.

## Methods

### Participants

All participants were Chinese and ranged in age from 6 to 18 years old; they were recruited from the First Hospital of Shanxi Medical University. In accordance with the Diagnostic and Statistical Manual of Mental Disorders, Fourth Edition (DSM-IV), at least two experienced psychiatrists independently made a consensus diagnosis of SCZ. All EOS patients should meet the criteria of the Positive and Negative Syndrome Scale (PANSS), obtaining a total score ≥ 60 and an IQ score ≥ 70. The exclusion criteria included organic diseases of the heart, liver, and kidneys; various immune diseases; brain damage; congenital brain malformations; brain tumors and epilepsy; mental retardation, and a history of taking antipsychotics, antimanic drugs, antidepressants, or mood stabilizers. We also excluded patients who were severely excited or impulsive. Healthy controls (HC) were age- and sex-matched and had never taken any medications within the past month. The exclusion criteria for HC were as follows: (1) met the criteria for patient inclusion or exclusion; (2) had a family history of any psychiatric or neurological disorder; (3) had a head injury or neonatal-related disorder; (4) had hyperpyretic convulsion before; and (5) was an adopted child or lived in a single-parent home.

Blood samples were collected from each participant early in the morning, before breakfast. Total RNA was isolated from peripheral blood mononuclear cells (PBMCs) using TRIzol (Invitrogen; USA) with on-column DNase I treatment as described by the manufacturer. Total RNA from each sample was quantified using a NanoDrop ND1000, and RNA integrity was assessed by standard denaturing agarose gel electrophoresis. An Agilent Array platform was used to perform microarray analysis.

**Fig. 1:**
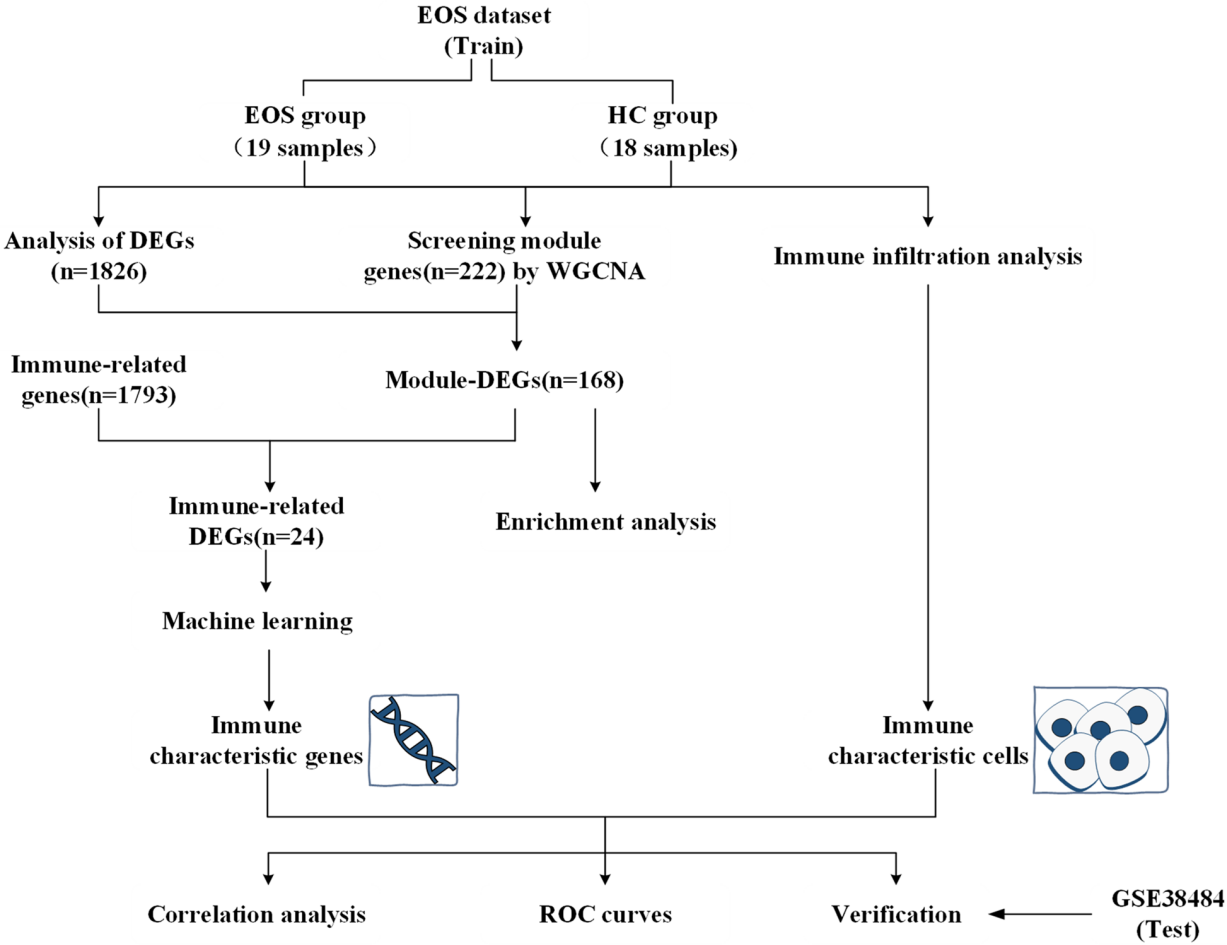
Flow chart. EOS, early-onset schizophrenia; HC, healthy controls; DEGs, differentially expressed genes; WGCNA, weighted gene co-expression network analysis; Module-DEGs, the intersection of the DEGs and the module hub genes; ROC, receiver operating characteristic

This study was approved by the Medical Research Ethics Committee of Shanxi Medical University (Ethical code: 2019-Y01). All experiments were performed according to approved guidelines. The adolescents and their parents or caregivers signed informed consent forms. Figure 1 depicts a flowchart of the study.

### Screening for immune characteristic genes

#### Data processing and screening for differentially expressed genes (DEGs)

We normalized the microarray data of the whole-genome mRNA expression array in the peripheral blood including 19 patients with EOS and 18 HC, using Log2. When multiple probes of Affymetrix corresponded to one gene, the mean value represented the horizontal expression of the gene.

We performed differential analysis of patients with EOS versus HC using the R software package Limma (version 3.40.6) to identify DEGs [20]. We set the screening criteria as differential multiples |log2 fold change (FC)|> 0.5, *p* < 0.05.

#### Weighted gene co-expression network analysis (WGCNA) and module gene selection

WGCNA is a systems biology method used to characterize correlation patterns between genes in microarray samples [21]. First, we determined the median absolute deviation (MAD) of each gene and removed genes with the smallest 50% MAD. Second, we filtered the DEG expression matrix using the goodSamplesGenes function. We also omitted unqualified genes and samples and constructed a scale-free co-expression network. Third, we calculated the adjacency relationship using the “soft” power threshold (β), derived from the co-expression similarity. We converted the adjacency relationship into a topological overlap matrix (TOM) to determine the gene ratio and dissimilarity. The fourth step entailed detecting the modules using hierarchical clustering and dynamic tree-cleavage functions. We classified genes with the same expression profiles into gene modules using mean-linked hierarchical clustering with a TOM-based dissimilarity index and a minimum genome size (n = 50) of the gene dendrogram, with a sensitivity of 3. Finally, we calculated the dissimilarity of the module’s characteristic genes and selected the cleavage line of the module’s dendrogram. Finally, we calculated the expression correlation of genes to obtain gene significance (GS). We computed the expression correlation of module eigenvectors and genes to obtain module membership (MM), and we screened for module hub genes based on the criteria (| MM |> 0.8 and | GS |> 0.1). The purpose of this analysis was to search for co-expressed gene modules, to investigate the relationship between EOS phenotype and module genes, and to identify candidate genes.

#### Screening for immune-related DEGs

We selected 168 module-DEGs considered to be correlated with EOS based on the intersection of the DEGs and module hub genes. We used the Immunology Database and Analysis Portal (ImmPort) (https://www.immport.org/) to download 1,793 immune-related genes [22]. Next, we selected 24 genes based on the intersection of the 168 module-DEGs and immune-related genes.

#### Selecting immune characteristic genes based on machine learning

To further screen for immune characteristic genes for diagnosis, we employed machine learning tools such as a support vector machine (SVM) and a random forest (RF); we did so using Wekemo Bioincloud (https://www.bioincloud.tech/) to screen for key genes. Furthermore, we integrated the data of gene expression, survival time, and survival with the “glmnet” R package, and we carried out regression analysis using the LASSO-Cox algorithm. Moreover, we optimized the model using threefold cross-validation. We selected the top 10 genes with the best classification effect using a SVM and RF, and we selected the intersections of the genes based on the SVM, RF, and LASSO-Cox algorithm.

A SVM is a classic supervised machine learning method; it uses the best “hyperplane” in a multidimensional space, which is created by multiple training features to categorize different classes [23]. It is a type of non-probability binary linear regression that is primarily applied to the problem of data classification in pattern recognition. Its basic model aims to find the best separation hyperplane in the feature space, such that the interval between the positive and negative samples is maximized on the training dataset. RF is an efficient decision tree algorithm that can be applied to sample classification and regression analyses [24]. It is a non-linear classifier that mines complex non-linear interdependencies between variables. Using a RF, it was possible to identify the key components that could distinguish between the two sets of samples. The LASSO-Cox algorithm is a regression method that combines variable selection and regularization, which can improve prediction accuracy [25].

### Functional enrichment analysis

The Kyoto Encyclopedia of Genes and Genomes (KEGG) database is widely used for systematic studies of gene function [26]. The Gene Ontology (GO) system provides structured and computable information regarding the functions of genes and gene products [27]. We performed functional enrichment analysis using the R-packet cluster analyzer and visualized the results using the Sangerbox platform (http://vip.sangerbox.com/) [28]. (FDR < 0.1 and *p*-value < 0.05).

### Immune infiltration analysis

We used CIBERSORT (https://cibersortx.stanford.edu/) to perform an immune infiltration analysis of the whole-genome mRNA expression array to identify characteristic immune cells. We employed Spearman’s correlation analysis to investigate the relationship between immune characteristic genes and cells.

CIBERSORT is a tool used to analyze the estimated immune cell infiltration based on linear support vector regression. It is employed to deconvolve the expression matrix of human immune cell subtypes to determine the types and functional states of different cells [29, 30].

### Identifying immunodiagnostic markers

#### Identifying the diagnostic efficacy of immune characteristic genes and cells

We used receiver operating characteristic (ROC) curve analysis to describe the discrimination accuracy of the diagnostic test or prediction model [31]. We constructed ROC curves to test the diagnostic efficacy of the immune characteristic genes and cells. In addition, we built a box map to visualize the expression of the immune characteristic genes.

#### Verifying the diagnostic efficacy of immune characteristic genes and cells

We employed GSE38484 as the test group and downloaded it from the Gene Expression Omnibus (GEO) datasets (https://www.ncbi.nlm.nih.gov/geo/). GSE38484 contains information on whole blood samples, which we collected from 106 patients with SCZ and 96 controls whose age was equal to or greater than 18. Supplementary Table 1 shows demographic information. We visualized the expression of immune characteristic genes and the proportion of immune characteristic cells using a box map. Subsequently, we built ROC curves to validate the diagnostic efficacy of the immune characteristic genes.

## Results

### Demographics and clinical characteristics of all participants

This study involved 19 EOS patients and 18 HC. There were no significant differences in sex or age between the EOS and the HC groups (Table 1).

**Table 1:** Demographics and clinical characteristics of all participants

	EOS	HC	*F*/* x*^2^	*p* value
n	19	18		
Age (years)	14.79 ± 1.90	15.67 ± 2.40	2.012	0.145^a^
Sex (M/F)	8/11	9/9	1.598	0.450^b^
PANSS total scores	62.17 ± 13.32			

*EOS* early-onset schizophrenia, *HC* healthy controls

^a^two-sample t-test

^b^two-sample chi-square test

### Screening for immune characteristic genes

#### Screening for DEGs

**Fig. 2:**
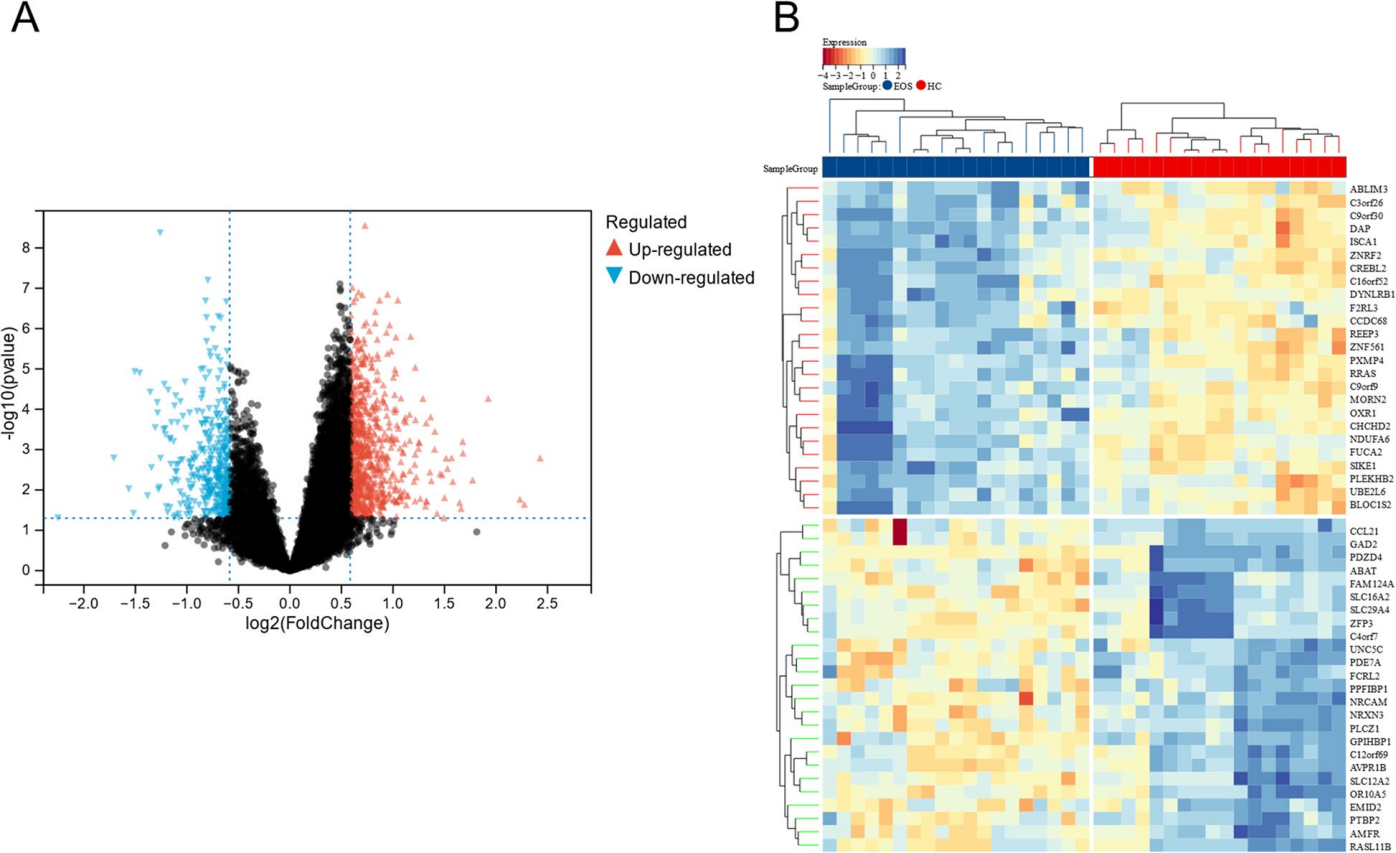
Differentially expressed gene analysis. **A** A volcanic map of 1,826 DEGs. The *X*-axis represents logFC and the *Y*-axis indicates -log10 (*p*-value). Red dots denote genes with logFC > 0.5 and *p* < 0.05. Blue dots refer to genes with logFC <  − 0.5 and *p* < 0.05. Black dots indicate genes with | logFC |< 0.5 and/or *p* > 0.05. **B** A heat map of 1,826 DEGs. The diagram presents the results of two-way hierarchical clustering of the top 25 DEGs and samples. The color in the heat map usually indicates the magnitude of the value, with red denoting a lower expression level and blue referring to a higher expression level. The darker the color, the larger the value

We identified 1,826 DEGs (in the EOS vs HC groups) using Limma analysis, of which 1,266 were upregulated and 560 were downregulated. Figure 2A and B depict volcanic and heat maps.

#### WGCNA and modular gene selection

**Fig. 3:**
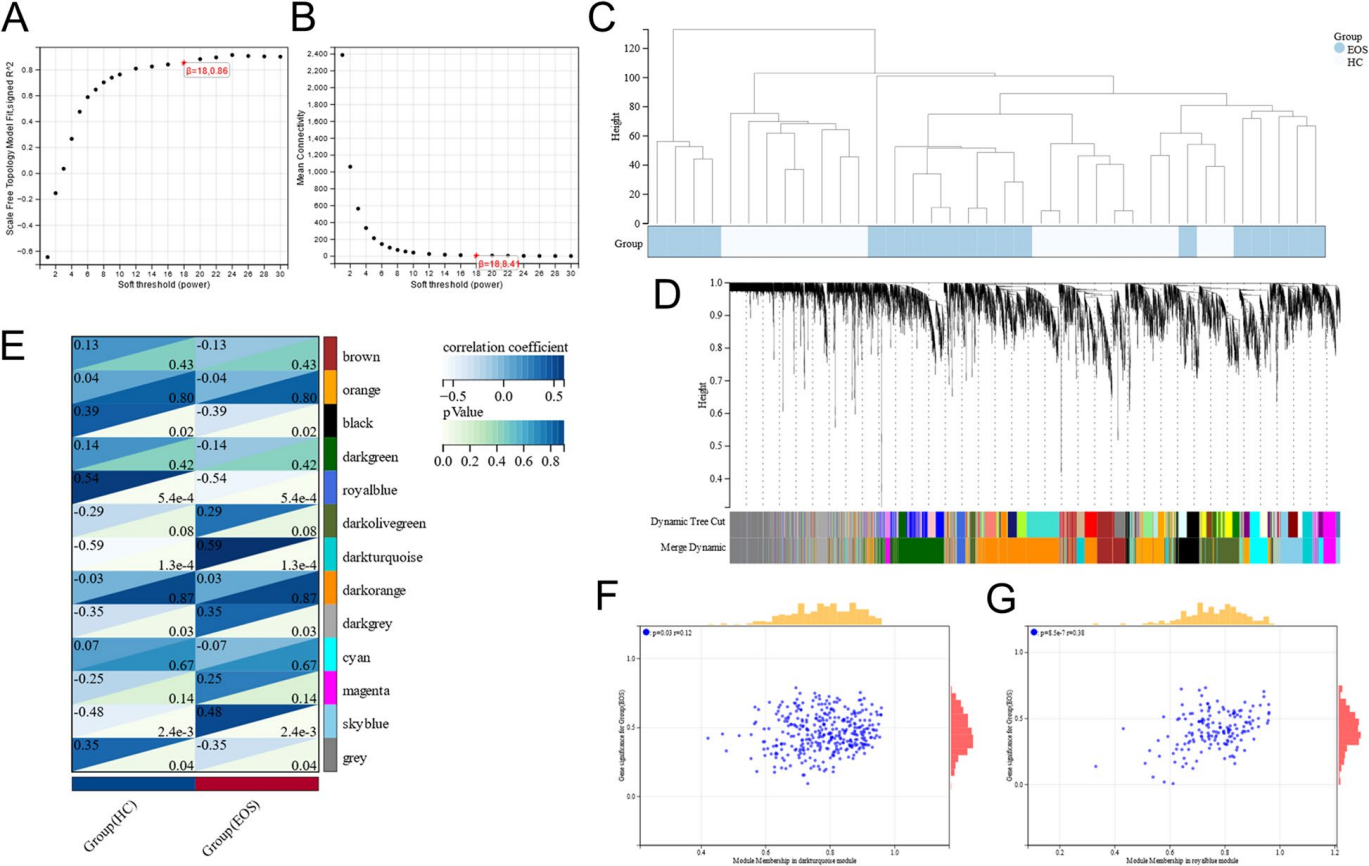
Results of WGCNA. **A** Determination of soft power threshold in the WGCNA. **B** Analysis of the scale-free index for various soft power thresholds (β). **C** Sample cluster dendrogram. Blue represents the EOS group and white indicates the HC group. **D** Hierarchical clustering tree diagram of module identification. Each color refers to a module. **E** A heat map of the correlation between the module eigengenes and clinical traits. We selected the royal blue and dark turquoise modules for subsequent analysis. **F** Correlation scatterplot of GS and GM in the dark turquoise module. **G** Correlation scatterplot of GS and GM in the royal blue module

We conducted WGCNA to identify the most relevant modules of EOS. We chose β = 18 (scale-free *R*^2^ = 0.86) as the clustering dendrogram according to scale independence and average connectivity (Fig. 3A and B). Figure 3C shows clustering dendrograms of the EOS and HC groups. We merged modules with distances of less than 0.25 and obtained 13 gene co-expression modules. These modules are displayed in Fig. 3D in different colors; the grey module is considered to a gene set that cannot be assigned to any module. Figure 3E presents the correlation between the modules and EOS. We selected modules with a correlation coefficient > 0.5. The royal blue (correlation coefficient = -0.54, *p* < 0.05) and dark turquoise modules (correlation coefficient = 0.59, *p* < 0.05) are correlated with EOS (Fig. 3E). The correlation scatterplots of GS and GM in the dark turquoise and royal blue modules (Fig. 3F and G) denote that MM and GS are positively correlated in the royal blue module (correlation coefficient = 0.38, *p* < 0.05); this suggests that the genes in the royal blue module are correlated with EOS and that they are important in this module.

We identified the royal blue and dark turquoise modules as key modules. According to the criteria (| MM |> 0.8 and | GS |> 0.1), 222 genes with connectivity in the key modules were clinically significant and we identified them as module genes.

#### Screening for immune-related DEGs

**Fig. 4:**
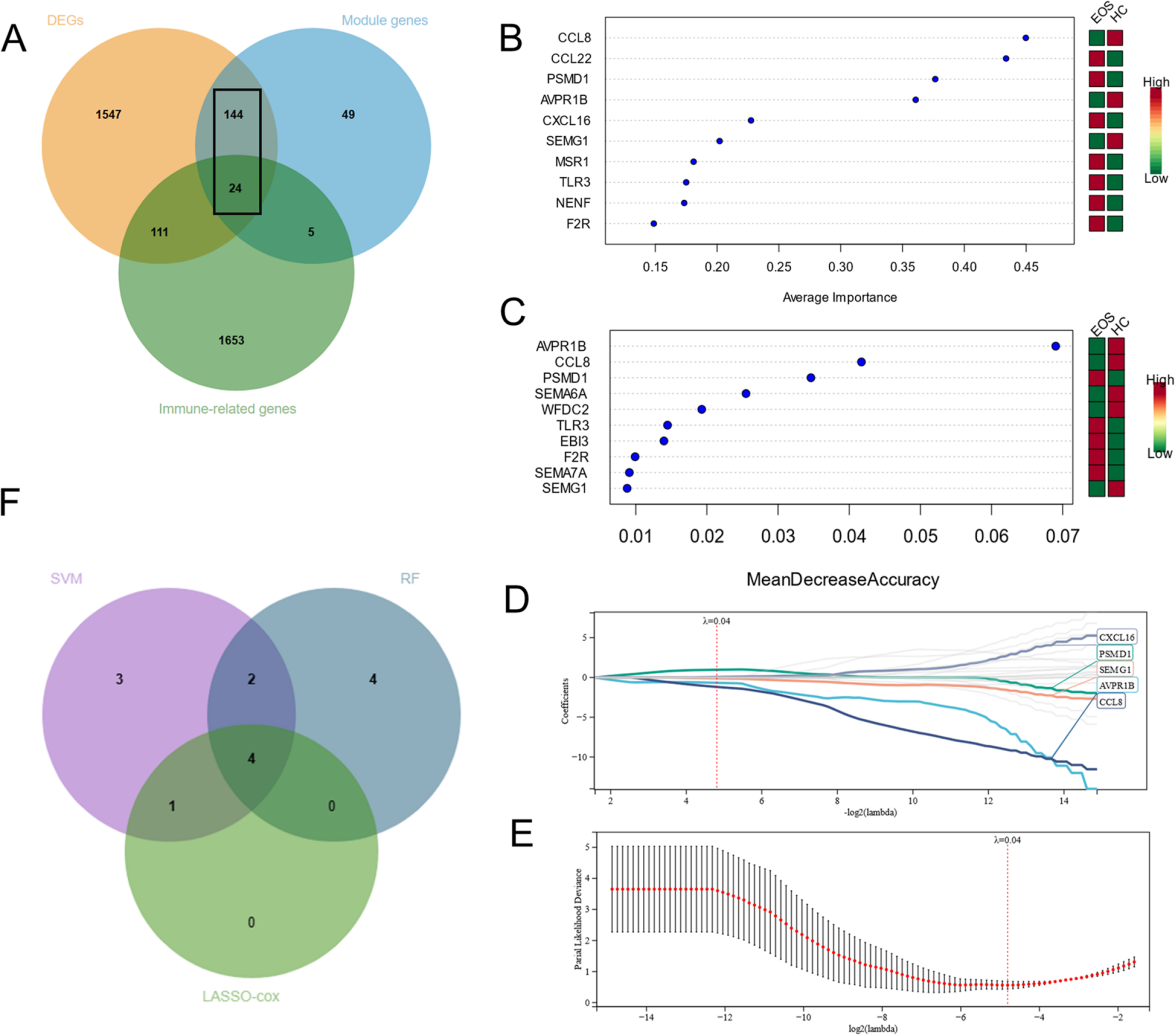
Screening for immune characteristic genes. **A** A Venn diagram for the DEGs, the module-DEGs and the immune-related genes. **B** We selected the top 10 genes, sorted by average importance, using a SVM. Red implies a higher expression level and green represents a lower expression level. **C** We selected the top 10 genes, sorted by mean decrease accuracy, using a RF. Red refers to a higher expression level and green denotes a lower expression level. **D**, **E** The key genes for screening using the LASSO-Cox algorithm. **F** A Venn diagram for the genes that we selected using a SVM, RF, and LASSO-Cox algorithm

We selected 168 module-DEGs based on the intersection of the DEGs and module genes, which we considered to have a correlation with EOS. We downloaded 1,793 immune-related genes from the ImmPort database. Next, we selected 24 immune-related DEGs based on the intersection of the 168 module-DEGs and immune-related genes (Fig. 4A).

#### Screening for immune characteristic genes using machine learning

To further screen for immune-related characteristic genes in terms of EOS, we selected the top 10 genes that could best classify EOS and HC using SVM and RF in machine learning (Fig. 4B, C). The LASSO-Cox regression outcomes revealed five key genes when we set the lambda value at a minimum of 0.0356731109826916 (Fig. 4D and E). We identified CCL8, PSMD1, AVPR1B, and SEMG1 as immune characteristic genes (Fig. 4F). Supplementary Table 2 outlines the biological significances among CCL8, PSMD1, AVPR1B and SEMG1.

### Functional enrichment analysis

**Fig. 5:**
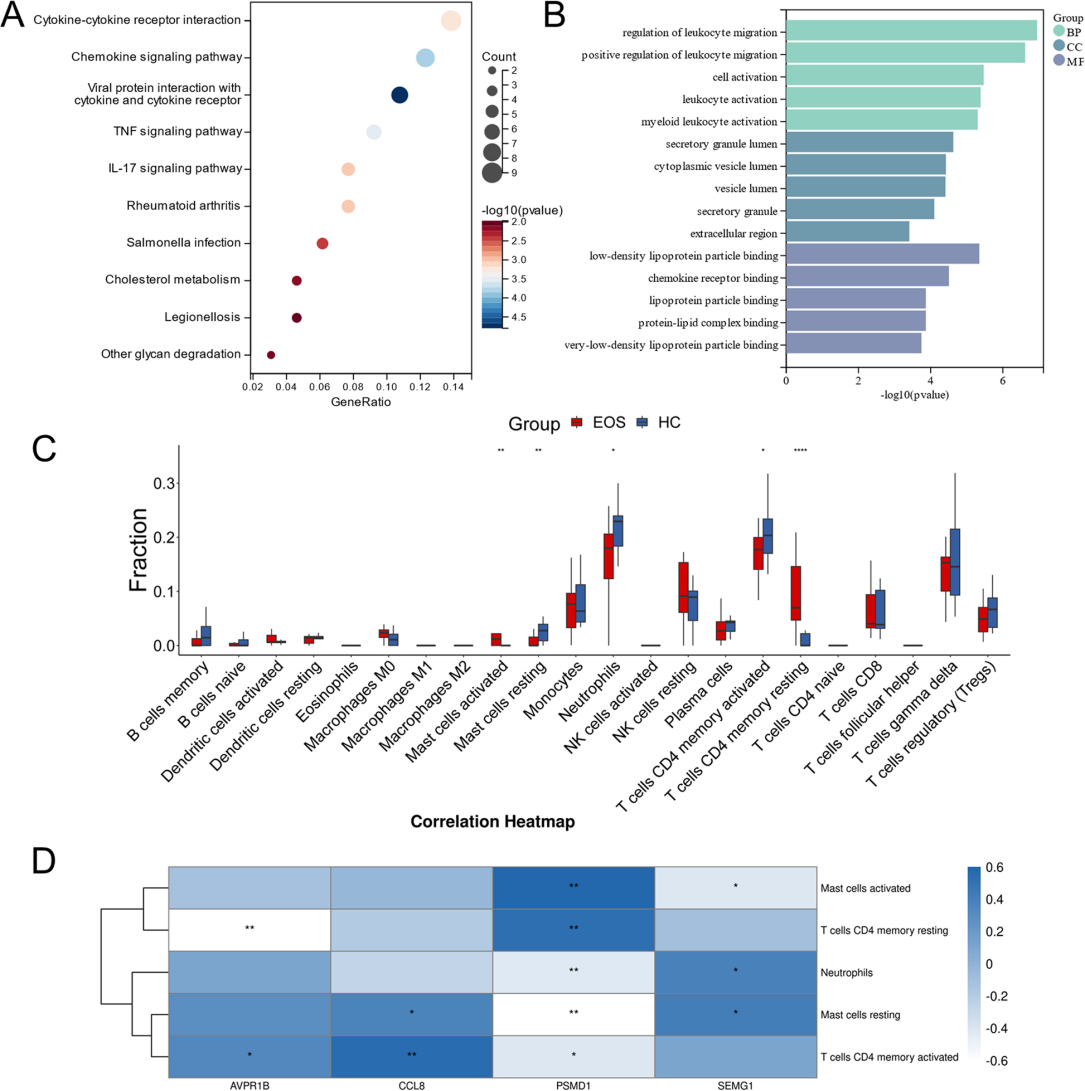
Results of analyzing functional enrichment and immune infiltration. **A** The top 10 pathways of KEGG enrichment analysis (FDR < 0.1, *p* < 0.05). **B** The top 5 pathways of biological processes, cellular components and molecular function of GO enrichment analysis (FDR < 0.1, *p* < 0.05). **C** The proportion of immune cells between EOS patients and HC. Red represents the EOS group and blue indicates the HC group. **D** Correlation between immune characteristic genes and cells. The color in the correlation heat map represents the magnitude of the relative coefficient. (^∗^*p* < 0.05, ^∗∗^*p* < 0.01, ^∗∗∗^*p* < 0.001)

To explore the pathogenesis and biological processes of EOS, we analyzed 168 module-DEGs for functional enrichment. Figure 5A depicts the outcomes of the KEGG enrichment analysis; they are mainly enriched in the chemokine signaling pathway, the TNF signaling pathway, the cytokine-cytokine receptor interaction, the IL-17 signaling pathway, rheumatoid arthritis, Salmonella infection and other pathways. We observed enrichment of 168 module-DEGs in 156 pathways, accounting for 46.99% of all pathways of enrichment of the 1,826 DEGs. Figure 5B displays the results of GO enrichment analysis: In biological processes (BPs), the main enrichment includes regulation, migration, activation, and chemotaxis of leukocytes. In cellular components (CCs), the chief enrichment occurs in secretory granules and vesicles. In molecular function (MF), the primary enrichment takes place in chemokine receptor binding, lipoprotein particle binding, and protein-lipid complex binding.

### Immune characteristic cells

We used the CIBERSORT algorithm to analyze immune infiltration and calculated the abundance of immune cells. Figure 5C portrays the ratio diagram of the 22 immune cell types in patients with EOS and in the HC. Compared with the HC, the proportion of activated mast cells and CD4^+^ memory resting T cells in EOS patients was significantly increased, and the proportion of resting mast cells, neutrophils, and CD4^+^ memory activated T cells was significantly decreased. We identified activated mast cells, CD4^+^ memory resting T cells, resting mast cells, neutrophils and CD4^+^ memory activated T cells as immune characteristic cells.

We used Spearman’s correlation analysis to explore the association between immune characteristic genes and cells. The results of correlation analysis indicated that AVPR1B was significantly and negatively correlated with CD4^+^ memory resting T cells (correlation coefficient = -0.61, *p* < 0.01); CCL8 was significantly and positively correlated with CD4^+^ memory activated T cells (correlation coefficient = 0.55, *p* < 0.01); PSMD1 was significantly and positively correlated with activated mast cells (correlation coefficient = 0.59, *p* < 0.01) and CD4^+^ memory resting T cells (correlation coefficient = 0.53, *p* < 0.01), and was significantly and negatively correlated with resting mast cells (correlation coefficient = -0.64, *p* < 0.01) (Fig. 5D).

### Immunodiagnostic markers

#### Identifying the diagnostic efficacy of immune characteristic genes and cells

**Fig. 6:**
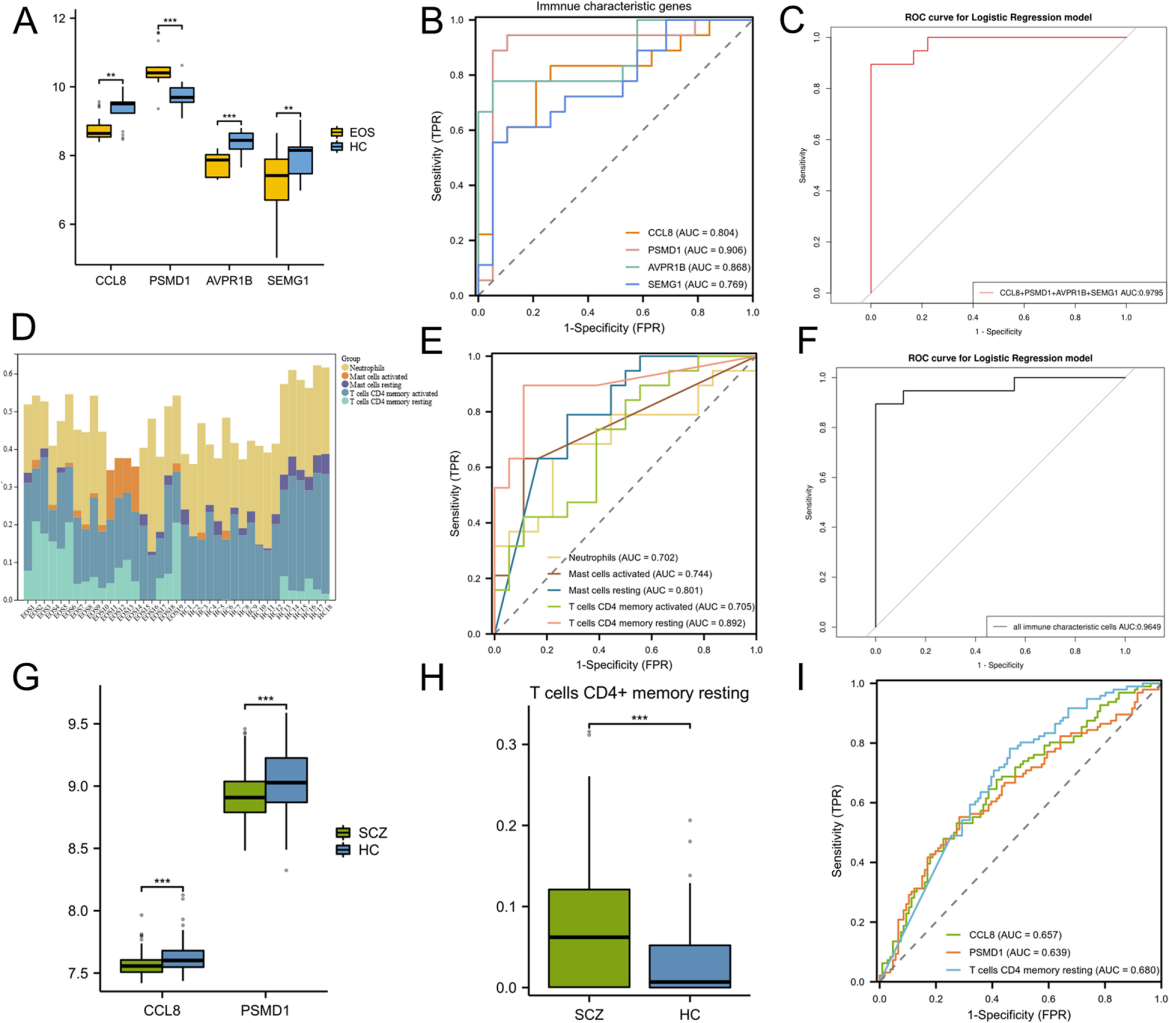
Results of ROC curves. **A** Expression profile analysis of CCL8, PSMD1, AVPR1B and SEMG1. Yellow denotes the EOS group and blue represents the HC group. **B** ROC curves of CCL8, PSMD1, AVPR1B and SEMG1. **C** ROC curve of the combination of CCL8, PSMD1, AVPR1B and SEMG1. **D** Stacked bar chart of immune characteristic cells. Each color indicates one kind of immune characteristic cell. **E** ROC curves of neutrophils, activated mast cells, resting mast cells, CD4 + memory activated T cells and CD4 + memory resting T cells. **F** ROC curve of the combination of neutrophils, activated mast cells, resting mast cells, CD4 + memory activated T cells and CD4 + memory resting T cells. **G** Expression profile analysis of CCL8 and PSMD1 of GSE38484. Green indicates the SCZ group and blue denotes the HC group. **H** The box-shaped diagram of the proportion of CD4 + memory resting T cells. Green represents the SCZ group and blue refers to the HC group. **I** ROC curves of CCL8, PSMD1 and CD4 + memory resting T cells of GSE38484. (^∗^*p* < 0.05, ^∗∗^*p* < 0.01, ^∗∗∗^*p* < 0.001)

We constructed a box-shaped diagram to visualize the expression of CCL8, PSMD1, AVPR1B, and SEMG1. The expression levels of PSMD1 in the EOS group were significantly higher than those in the HC group, whereas the expression levels of CCL8, AVPR1B, and SEMG1 in the EOS group were significantly lower than those in the HC group (Fig. 6A). We established ROC curves to evaluate the diagnostic efficacy of the immune characteristic genes—CCL8: AUC = 0.804, PSMD1: AUC = 0.906, AVPR1B: AUC = 0.868, SEMG1: AUC = 0.769, and the combination of CCL8, PSMD1, AVPR1B and SEMG1: AUC = 0.9795 (Fig. 6B and C).

The stacked bar chart of immune characteristic cells shows the proportions of neutrophils, activated mast cells, resting mast cells, CD4^+^ memory activated T cells and CD4^+^ memory resting T cells in patients with EOS and the HC (Fig. 6D). ROC curve analysis showed the diagnostic efficacy of immune characteristic cells, neutrophils: AUC = 0.702, activated mast cells: AUC = 0.744, resting mast cells: AUC = 0.801, CD4^+^ memory activated T cells: AUC = 0.705, CD4^+^ memory resting T cells: AUC = 0.829, and the combination of immune characteristic cells: AUC = 0.9649 (Fig. 6E and F).

#### Verifying the diagnostic efficacy of immune characteristic genes

In GSE38484, the expression of CCL8 and PSMD1 in patients with SCZ was lower than that in the controls (Fig. 6G). The proportion of CD4^+^ memory resting T cells was higher than that in the controls (Fig. 6H). However, there was no significant difference between patients with SCZ and controls, not only in the expression of AVPR1B and SEMG1, but also in the proportion of neutrophils, activated mast cells, resting mast cells and CD4^+^ memory activated T cells. ROC curve analysis revealed that CCL8, PSMD1 and CD4^+^ memory resting T cells also had good diagnostic efficacy in GSE38484, CCL8: AUC = 0.657, PSMD1: AUC = 0.639, CD4^+^ memory resting T cells: AUC = 0.680 (Fig. 6I).

## Discussion

Our findings suggest that immunological dysfunction is involved in SCZ progression. In addition, we identified immune characteristic genes and cells. CCL8, PSMD1, AVPR1B and SEMG1 may regulate EOS peripheral immune cells in EOS. Moreover, immune characteristic genes and cells have high diagnostic efficiency. Hence, these miRNAs may serve as diagnostic markers and therapeutic targets.

CCL8 (chemokine ligand 8) is a protein-coding gene, which enrichment analysis revealed is involved in regulating the chemokine signaling pathway, the cytokine-cytokine receptor interaction, the regulation of leukocytes, migration, chemotaxis and other BPs. CCL8 is involved in the immune regulation and inflammatory processes. In our study, the expression of CCL8 in the peripheral blood was lower than that in the controls in both the EOS and SCZ groups. One study found that the level of CCL8 in the cerebrospinal fluid of patients was significantly higher than that in the controls, which is contrary to our findings [32]. The heterogeneity of the results may be related to inconsistencies in age, race, detection method, small sample sizes, and differences in sample types. CCL8 promotes the chemotaxis of pro-inflammatory cells in SCZ, which is closely associated with the pathogenesis of psychiatric disorders [33]. PSMD1 (proteasome 26S subunit, non-ATPase 1) encodes the constituent components of the proteasome, and is involved in cell proliferation, invasion, and migration [34]. AVPR1B (arginine vasopressin receptor 1B) encodes the arginine vasopressin receptor; however, its function is unknown. Genetic variants of AVPR1B are linked to psychiatric disorders commonly diagnosed in childhood, including autism, attention deficit hyperactivity disorder (ADHD), and mood disorders [35–37]. SEMG1 (semen coagulation protein), encodes the main protein in semen and is related to male fertility [38].

The outcomes of the KEGG enrichment analysis demonstrated that the 168 module-DEGs were primarily enriched in the chemokine signaling pathway, the TNF signaling pathway, the cytokine-cytokine receptor interaction, and the IL-17 signaling pathway. These may be the regulatory pathways involved in the pathophysiological mechanisms of SCZ. The chemokine signaling pathways regulate cell migration, participate in mediating immune cell homeostasis in healthy peripheral tissues, and maintain immune tolerance [39, 40]. The TNF signaling pathway mediates hematopoiesis, immune surveillance, tumor regression and infection prevention [41]. The cytokine-cytokine receptor interaction pathway mediates the development and differentiation, immune response and regulation of immune cells. The IL-17 signaling pathway mediates various processes such as host defense, tissue repair, inflammatory disease pathogenesis and cancer progression [42]. The GO enrichment analysis indicated that the DEGs were mostly enriched via leukocyte regulation, migration, activation, chemotaxis, and chemokine receptor binding. These pathways are involved in regulating the immune system and indirectly confirm the immune-inflammatory hypothesis.

In this study, we observed functional activation of mast cells and hypofunctionality of CD4 + memory T cells and neutrophils. CD4 + memory T cells have memory functions and can differentiate into T-helper 1 (Th1), Th2, Th-17, and regulatory T cells (Tregs) for immunoregulation during reinfection [43]. Tregs are key immunoregulatory cells involved in controlling inflammatory processes, and their functions are directly linked to the human leukocyte antigen (HLA) gene, which has been associated with genetic studies of SCZ [44, 45]. In our study, we found that CCL8, PSMD1, AVPR1B and SEMG1 significantly correlated with mast cells and CD4 + memory T cells. Moreover, 168 module-DEGs were enriched in the chemokine and IL-17 signaling pathways. Therefore, we speculated that CCL8, PSMD1, AVPR1B and SEMG1 may have potential regulatory effects on EOS peripheral immune cells via the chemokine and IL-17 signaling pathways.

The AUC values of the immune characteristic genes and c cells were greater than 0.7, indicating good diagnostic efficacy. We found that combining immune characteristic genes or cells can further improve the accuracy of diagnosis, and they are expected to become peripheral blood biomarkers for the early diagnosis of EOS. In GSE38484, the expression of CCL8 in patients with SCZ was lower than that in the controls. In addition, the proportion of CD4^+^ memory resting T cells was higher than that in the controls, which is consistent with our results in the training group. However, the expression of PSMD1 in patients with SCZ was lower than that in the controls, and the expressions of AVPR1B and SEMG1 were not significantly different between patients with SCZ and the controls. These results are inconsistent with those of the training group. However, this may be related to inconsistencies in patients’ ages, sex, a small sample size, and differences in sample types.

Our study has several limitations. First, the number of patients included in this study was small. A larger sample size is required to verify the findings. Second, our study was a cross-sectional study, therefore it is impossible to infer the mechanism between immune characteristic genes and cells. Finally, it remains to be seen whether the expression of immune characteristic genes and cells can be used as biomarkers for early diagnosis and the prediction of therapeutic effects.

## Conclusion

This study revealed immune characteristic genes and cells of EOS, further verifying that immune system function is altered in SCZ, and that CCL8, PSMD1, AVPR1B, SEMG1, activated mast cells, resting mast cells, neutrophils, CD4^+^ memory resting T cells and CD4^+^ memory activated T cells may be EOS biomarkers for the diagnosis and prediction of therapeutic efficacy.

### Supplementary Information

**Supplementary Material 1.**

## Data Availability

The original contributions presented in the study are included in the article, further inquiries can be directed to the corresponding author. GSE38484 was retrieved from the GEO data repository [https://www.ncbi.nlm.nih.gov/geo/query/acc.cgi?acc=GSE38484].

## Abbreviations

SCZ: Schizophrenia
EOS: Early-onset schizophrenia
CNS: Central nervous system
DSM-IV: Diagnostic and Statistical Manual of Mental Disorders, Fourth Edition
PANSS: Positive and Negative Syndrome Scale
HC: Healthy controls
PBMCs: Peripheral blood mononuclear cells
DEGs: Differentially expressed genes
WGCNA: Weighted gene co-expression network analysis
MAD: Median absolute deviation
TOM: Topological overlap matrix
GS: Gene significance
MM: Module membership
SVM: Support vector machine
RF: Random forest
KEGG: Kyoto Encyclopedia of Genes and Genomes
GO: Gene Ontology
ROC: Receiver operating characteristic
GEO: Gene Expression Omnibus
Th1: T-helper 1
Tregs: Regulatory T cells
HLA: Human leucocyte antigen

## References

[1] Mueser KT, McGurk SR (2004). Schizophrenia. Lancet Lond Engl.

[2] Zwicker A, Denovan-Wright EM, Uher R (2018). Gene-environment interplay in the etiology of psychosis. Psychol Med.

[3] Kumra S, Charles SS (2008). Editorial: research progress in early-onset schizophrenia. Schizophr Bull.

[4] Khandaker GM, Cousins L, Deakin J, Lennox BR, Yolken R, Jones PB (2015). Inflammation and immunity in schizophrenia: implications for pathophysiology and treatment. Lancet Psychiatry.

[5] Müller N (2018). Inflammation in schizophrenia: pathogenetic aspects and therapeutic considerations. Schizophr Bull.

[6] Paquin V, Lapierre M, Veru F, King S (2021). Early environmental upheaval and the risk for schizophrenia. Annu Rev Clin Psychol.

[7] Müller N, Schwarz MJ (2010). Immune system and schizophrenia. Curr Immunol Rev.

[8] Potvin S, Stip E, Sepehry AA, Gendron A, Bah R, Kouassi E (2008). Inflammatory cytokine alterations in schizophrenia: a systematic quantitative review. Biol Psychiatry.

[9] Savitz J, Harrison NA (2018). Interoception and inflammation in psychiatric disorders. Biol Psychiatry Cogn Neurosci Neuroimaging.

[10] Schizophrenia Working Group of the Psychiatric Genomics Consortium (2014). Biological insights from 108 schizophrenia-associated genetic loci. Nature.

[11] North HF, Weissleder C, Fullerton JM, Sager R, Webster MJ, Weickert CS (2021). A schizophrenia subgroup with elevated inflammation displays reduced microglia, increased peripheral immune cell and altered neurogenesis marker gene expression in the subependymal zone. Transl Psychiatry.

[12] North HF, Weissleder C, Fullerton JM, Webster MJ, Weickert CS (2022). Increased immune cell and altered microglia and neurogenesis transcripts in an Australian schizophrenia subgroup with elevated inflammation. Schizophr Res.

[13] Fernandez-Egea E, Vértes PE, Flint SM, Turner L, Mustafa S, Hatton A (2016). peripheral immune cell populations associated with cognitive deficits and negative symptoms of treatment-resistant schizophrenia. PLoS One.

[14] Du Y, Gao Y, Wu G, Li Z, Du X, Li J (2022). Exploration of the relationship between hippocampus and immune system in schizophrenia based on immune infiltration analysis. Front Immunol..

[15] Harris LW, Pietsch S, Cheng TMK, Schwarz E, Guest PC, Bahn S (2012). Comparison of Peripheral and Central Schizophrenia Biomarker Profiles. PLoS One..

[16] Cao T, Li N, Cai H (2020). Candidate metabolic biomarkers for schizophrenia in CNS and periphery: do any possible associations exist?. Schizophr Res.

[17] Soares-Weiser K, Maayan N, Bergman H, Davenport C, Kirkham AJ, Grabowski S (2015). First rank symptoms for schizophrenia. Cochrane Database Syst Rev..

[18] Chan MK, Guest PC, Levin Y, Umrania Y, Schwarz E, Bahn S, et al. Converging evidence of blood-based biomarkers for schizophrenia: an update. Int Rev Neurobiol. 2011;101:95–144. 10.1016/B978-0-12-387718-5.00005-5 22050850

[19] R K, A S, Dk L, R G, B S (2021). Prospecting the intricate role of novel and potent biomarkers in schizophrenia. Curr Top Med Chem..

[20] Ritchie ME, Phipson B, Wu D, Hu Y, Law CW, Shi W (2015). limma powers differential expression analyses for RNA-sequencing and microarray studies. Nucleic Acids Res.

[21] Langfelder P, Horvath S (2008). WGCNA: an R package for weighted correlation network analysis. BMC Bioinformatics.

[22] Bhattacharya S, Dunn P, Thomas CG, Smith B, Schaefer H, Chen J (2018). ImmPort, toward repurposing of open access immunological assay data for translational and clinical research. Sci Data.

[23] Winters-Hilt S, Yelundur A, McChesney C, Landry M (2006). Support vector machine implementations for classification & clustering. BMC Bioinformatics.

[24] Rigatti SJ (2017). Random forest. J Insur Med N Y N.

[25] Tibshirani R (1997). The lasso method for variable selection in the Cox model. Stat Med.

[26] Kanehisa M, Goto S (2000). KEGG: Kyoto encyclopedia of genes and genomes. Nucleic Acids Res.

[27] Gene Ontology Consortium (2015). Gene Ontology Consortium: going forward. Nucleic Acids Res..

[28] Shen W, Song Z, Zhong X, Huang M, Shen D, Gao P (2022). Sangerbox: a comprehensive, interaction-friendly clinical bioinformatics analysis platform. iMeta..

[29] Newman AM, Liu CL, Green MR, Gentles AJ, Feng W, Xu Y (2015). Robust enumeration of cell subsets from tissue expression profiles. Nat Methods.

[30] Chen B, Khodadoust MS, Liu CL, Newman AM, Alizadeh AA (2018). Profiling tumor infiltrating immune cells with CIBERSORT. Methods Mol Biol Clifton NJ.

[31] Obuchowski NA, Bullen JA (2018). Receiver operating characteristic (ROC) curves: review of methods with applications in diagnostic medicine. Phys Med Biol..

[32] Hayes LN, Severance EG, Leek JT, Gressitt KL, Rohleder C, Coughlin JM (2014). Inflammatory molecular signature associated with infectious agents in psychosis. Schizophr Bull.

[33] Stuart MJ, Singhal G, Baune BT (2015). Systematic review of the neurobiological relevance of chemokines to psychiatric disorders. Front Cell Neurosci.

[34] Li Y, Liu X, Zhao F, Zhao Z, Li X, Wang J, et al. Comprehensive analysis of PSMD family members and validation of PSMD9 as a potential therapeutic target in human glioblastoma. CNS Neurosci Ther. 2024;30:e14366. 10.1111/cns.14366 PMC10848081 37485655

[35] Verhagen M, Verweij KJH, Lodder GMA, Goossens L, Verschueren K, Van Leeuwen K (2020). A SNP, gene, and polygenic risk score approach of oxytocin-vasopressin genes in adolescents’ loneliness. J Res Adolesc Off J Soc Res Adolesc..

[36] Dempster EL, Burcescu I, Wigg K, Kiss E, Baji I, Gadoros J (2007). Evidence of an association between the vasopressin V1b receptor gene (AVPR1B) and childhood-onset mood disorders. Arch Gen Psychiatry.

[37] van West D, Del-Favero J, Deboutte D, Van Broeckhoven C, Claes S (2010). Associations between common arginine vasopressin 1b receptor and glucocorticoid receptor gene variants and HPA axis responses to psychosocial stress in a child psychiatric population. Psychiatry Res.

[38] Yu Q, Zhou Q, Wei Q, Li J, Feng C, Mao X (2014). SEMG1 may be the candidate gene for idiopathic asthenozoospermia. Andrologia.

[39] Wang J, Knaut H (2014). Chemokine signaling in development and disease. Dev Camb Engl.

[40] Chen D, Zhang X, Li Z, Zhu B (2021). Metabolic regulatory crosstalk between tumor microenvironment and tumor-associated macrophages. Theranostics.

[41] Aggarwal BB (2003). Signalling pathways of the TNF superfamily: a double-edged sword. Nat Rev Immunol.

[42] Li X, Bechara R, Zhao J, McGeachy MJ, Gaffen SL (2019). IL-17 receptor-based signaling and implications for disease. Nat Immunol.

[43] Stockinger B, Bourgeois C, Kassiotis G (2006). CD4+ memory T cells: functional differentiation and homeostasis. Immunol Rev.

[44] Kelly DL, Li X, Kilday C, Feldman S, Clark S, Liu F (2018). Increased circulating regulatory T cells in medicated people with schizophrenia. Psychiatry Res.

[45] Corsi-Zuelli F, Deakin B, de Lima MHF, Qureshi O, Barnes NM, Upthegrove R (2021). T regulatory cells as a potential therapeutic target in psychosis? Current challenges and future perspectives. Brain Behav Immun Health.

