# Supplementary material for: The role of the immune system in early-onset schizophrenia: identifying immune characteristic genes and cells from peripheral blood
Source: BMC Immunol. 2024 May 3;25:26. doi: 10.1186/s12865-024-00618-y (PMC11067251; doi:10.1186/s12865-024-00618-y)
Supplement: Supplementary file 1 — Supplementary Material 1. [file 12865_2024_618_MOESM1_ESM.docx]

**Attentional materials:**

Supplementary Table 1

Demographic information of GSE38484

| **Characteristics** | **SCZ(n=106)** | **Controls (n=96)** | ***p* value** |
| --- | --- | --- | --- |
| **Age，years** |  |  | 0.879^a^ |
| Mean ± sd | 39.58 ± 10.74 | 39.31 ± 14.19 |  |
| Median[min-max] | 39.58 [18, 63] | 39.31 [18, 81] |  |
| **Sex** |  |  | 0.000^b^ |
| Female | 30(14.85%) | 54(26.73%) |  |
| Male | 76(37.62%) | 42(20.79%) |  |

a: Two-sample t-test; b: two-sample Chi-square test; SCZ, schizophrenia; sd, standard deviation.

Supplementary Table 2

Biological significance of immune characteristic genes

| **Gene abbreviation** | **Full name** | **Biological significance** |
| --- | --- | --- |
| CCL8 | chemokine ligand 8 | Chemotactic factor that attracts monocytes, lymphocytes, basophils and eosinophils. May play a role in neoplasia and inflammatory host responses. This protein can bind heparin. The processed form MCP-2(6-76) does not show monocyte chemotactic activity, but inhibits the chemotactic effect most predominantly of CCL7, and also of CCL2 and CCL5 and CCL8. |
| PSMD1 | proteasome 26S subunit, non-ATPase 1 | Component of the 26S proteasome, a multiprotein complex involved in the ATP-dependent degradation of ubiquitinated proteins. This complex plays a key role in the maintenance of protein homeostasis by removing misfolded or damaged proteins, which could impair cellular functions, and by removing proteins whose functions are no longer required. Therefore, the proteasome participates in numerous cellular processes, including cell cycle progression, apoptosis, or DNA damage repair. |
| AVPR1B | arginine vasopressin receptor 1B | The protein encoded by this gene acts as receptor for arginine vasopressin. This receptor belongs to the subfamily of G-protein coupled receptors which includes AVPR1A, V2R and OXT receptors. Its activity is mediated by G proteins which stimulate a phosphatidylinositol-calcium second messenger system. The receptor is primarily located in the anterior pituitary, where it stimulates ACTH release. It is expressed at high levels in ACTH-secreting pituitary adenomas as well as in bronchial carcinoids responsible for the ectopic ACTH syndrome. A spliced antisense transcript of this gene has been reported but its function is not known. |
| SEMG1 | semen coagulation protein | The protein encoded by this gene is the predominant protein in semen. The encoded secreted protein is involved in the formation of a gel matrix that encases ejaculated spermatozoa. This preproprotein is proteolytically processed by the prostate-specific antigen (PSA) protease to generate multiple peptide products that exhibit distinct functions. One of these peptides, SgI-29, is an antimicrobial peptide with antibacterial activity. This proteolysis process also breaks down the gel matrix and allows the spermatozoa to move more freely. |
